# Assessment of the Parameters of Oxidative Stress Depending on the Metabolic and Anthropometric Status Indicators in Women with PCOS

**DOI:** 10.3390/life12020225

**Published:** 2022-01-31

**Authors:** Jolanta Nawrocka-Rutkowska, Iwona Szydłowska, Katarzyna Jakubowska, Maria Olszewska, Dariusz Chlubek, Aleksandra Rył, Małgorzata Szczuko, Andrzej Starczewski

**Affiliations:** 1 Department of Gynecology, Endocrinology and Gynecological Oncology, Pomeranian Medical University in Szczecin, 71-252 Szczecin, Poland; iwona.szydlowska@pum.edu.pl (I.S.); andrzejstarcz@o2.pl (A.S.); 2 Department of Biochemistry and Medical Chemistry, Pomeranian Medical University in Szczecin, 70-111 Szczecin, Poland; jakubkas@pum.edu.pl (K.J.); marolsz@pum.edu.pl (M.O.); dchlubek@pum.edu.pl (D.C.); 3 Department of Medical Rehabilitation and Clinical Physiotherapy, Pomeranian Medical University in Szczecin, 71-210 Szczecin, Poland; aleksandra.ryl@pum.edu.pl; 4 Department of Human Nutrition and Metabolomics, Pomeranian Medical University in Szczecin, 71-460 Szczecin, Poland; malgorzata.szczuko@pum.edu.pl

**Keywords:** PCOS, oxidative stress, insulin resistance, glutathione peroxidase (GPx), catalase (CAT), superoxide dismutase (SOD), malonodialdehyde (MDA)

## Abstract

Polycystic ovary syndrome (PCOS) is one of the most common endocrinopathies in females of reproductive age. In women with PCOS, metabolic disorders such as insulin resistance (IR), hyperinsulinemia, obesity, diabetes mellitus, and other elements of metabolic syndrome are likely to occur. Studies have shown an increase in the concentration and activity of oxidative stress (OS) markers in patients with PCOS, compared to that in unaffected women. The aim of this study was to evaluate the parameters of OS in PCOS and their activity in relation to women without menstrual disorders with a normal body weight. Then, we compared malonodialdehyde (MDA), superoxide dismutase (SOD), catalase (CAT), and glutathione peroxidase (GPx), including overweight and obesity, hyperandrogenemia, and IR in the PCOS group. The study included 35 women aged 18–46, hospitalized for menstrual disorders in the form of infrequent menstruation. In 26 women, PCOS was diagnosed on the basis of the Rotterdam Criteria; these patients qualified for the study group. The control group (*n* = 21) consisted of patients without menstrual disorders and without PCOS in an ultrasound examination. Patients were diagnosed between the 2nd and 5th day of the cycle. The parameters of OS were analyzed and compared with the anthropometric parameters and the lipid profile of the patients. Enzymatic activity of GPx, CAT, SOD, and MDA levels was determined in both groups. MDA levels and CAT activity differed significantly between the groups. There was a decrease in MDA levels in the IR group and the involvement of GPx in the excess weight and obesity and IR group accompanied by an increase in hip circumference. It therefore seems that IR may be the main risk factor to exposure to OS in patients with PCOS, independent from obesity. In addition, GPx is involved in every step in the development of the pathological condition in PCOS.

## 1. Introduction

Polycystic ovary syndrome (PCOS) is one of the most common endocrinopathies at reproductive age and may affect 5–14% of women [1,2]. The etiology of PCOS is multifactorial; therefore, the various aspects of this disease are still the subjects of research. Women with PCOS are likely to develop metabolic disorders such as insulin resistance (IR), hyperinsulinemia, obesity (especially abdominally), diabetes mellitus, and other elements of metabolic syndrome [3].

One of the most common abnormalities observed in PCOS is hyperandrogenism. Clinically, it manifests itself mainly in hirsutism, but also sometimes a severe, difficult-to-treat form of acne [4]. Excessive production of androgens can onset at prenatal age, resulting in android-type abdominal obesity [5]. Hyperandrogenemia is also exacerbated by hyperinsulinemia; decreased concentrations of the sex hormone binding globulin (SHBG) cause a large pool of androgens to circulate in a free biologically active form. Irregular menstruation, infertility, and loss of pregnancies, as well as far-reaching consequences of metabolic disorders, such as arterial hypertension, cardiovascular disorders, and estrogen-dependent cancers, cause multiple health and emotional difficulties for PCOS patients at every stage of their lives [6,7,8,9]. IR is regarded as the main mechanism of PCOS pathogenesis and occurs in approximately 50–70% of PCOS women. IR markers such as homeostatic model assessment—insulin resistance (HOMA-IR), are significantly increased in PCOS patients compared with women without PCOS [10,11]. It is estimated that about 90% of obese women with PCOS and 75% of lean women with PCOS have a tendency toward the occurrence of dysfunction of the blood system associated with atherosclerosis, aging, obesity, type 2 diabetes, cardiovascular disease, and cancer [12].

Excess weight and obesity disrupt ovulation and cause and/or aggravate IR and hyperandrogenism. Progressive obesity-related disorders manifest in increased adipogenesis and decreased lipolysis. Interactions between adipose tissue and ovarian function result in folliculogenesis disorders. Another consequence of obesity is the secretion of proinflammatory adipokines, which by affecting the thecal cells, have an impact on the production of androgens in the ovaries [13,14]. Excessive production of androgens in the ovarian thecal cells is thought to be caused by constant acyclic stimulation by the luteinizing hormone (LH), whereas a relative deficiency of follicle-stimulating hormone (FSH) is responsible for a chronic lack of ovulation [9]. Ovarian hyperandrogenism results in defects in follicle selection, maturation, and ovulation. The lack of ovulation triggers menstrual disorders and infertility, as the result of a deficiency in gestagens and relative hyperestrogenism. This increases the risk of estrogen-dependent cancers [7].

A number of studies point toward the significant role chronic inflammation plays in PCOS. It can cause adipocyte hypertrophy and, consequently, tissue hypoxia. Inflammation goes hand in hand with obesity and hyperinsulinemia with excess androgen levels [15]. Metabolic disorders in PCOS cause an increase in the number of free radicals arising from genetic and environmental conditions, which causes increased OS and the development of inflammation [16]. Inflammation has been reported to be associated with IR in PCOS [11].

OS, as well as hyperinsulinism and obesity, worsen glucose metabolism, namely its uptake by skeletal muscles and adipose tissue. Increased insulin levels secondary to IR may accelerate protein oxidation independent from hyperglycemia [12]. According to some authors, implementation of antioxidant therapy can improve tissue sensitivity to insulin [17]. IR correlates with OS parameters. Hyperglycemia and higher levels of free fatty acid lead to the production of reactive oxygen species (ROS) [10,11]. OS is defined as an imbalance of presently produced oxidants and antioxidants [18]. At low concentrations, reactive forms of oxygen and nitrogen can constitute an effective defense against infectious agents; in excess, however, they cause damage to deoxyribonucleic acid (DNA), cellular lipids, and proteins [19]. Thus, there are a number of mechanisms that neutralize oxidizing substances in the body. These are macromolecular antioxidants, which include MDA and enzymes such as: SOD, CAT, and GPx, and low-molecular-weight ones: ferritin, albumin, glutathione, vitamin E, and ascorbic acid. Ascorbic acid is the first line of antioxidant defense of the organism, and thus, it also protects other antioxidants such as vitamin E.

Peroxidases catalyze hydrogen peroxide reduction reactions, and catalases decompose hydrogen peroxide. Peroxide accumulation can lead to the formation of free radicals. Free radicals have the ability to damage cell membranes and can contribute to atherosclerosis and cancer. Peroxidase, together with vitamin E, constitutes a part of the anti-lipid oxidation protective system [20].

CAT is an enzyme involved in the elimination of free radicals. Reduced CAT activity indicates the presence of long-term OS [21,22].

MDA is another marker of chronic OS. Lipid peroxidation of polyunsaturated fatty acids generates MDA [23]. Its concentration increases under conditions of the heightened production of ROS. MDA is used as an indicator in assessing the effectiveness of antioxidant therapy.

A decrease in concentrations of OS parameters can be achieved by weight reduction attained through a diet rich in antioxidants and/or a diet with reduced calorie count and lowered glycemic index (GI) [6,8,24]. Although studies confirmed an increase in the concentration and activity of OS markers in patients with PCOS compared to women without the disease, it is not entirely clear why this occurs.

It is assumed that oxidative stress-inducing factors in PCOS include hyperandrogenemia, genetically and/or environmentally conditioned IR, and obesity, especially abdominal. OS can also be caused by infections. This thesis is not very popular, but it is possible. Whether these factors induce OS to a similar degree remains unknown [8,16,24]. The evaluation of OS and antioxidant biomarkers have been suggested as useful tools in estimating the risk of oxidative damage and associated diseases, which can be helpful in the prevention and management of oxidative diseases [25].

The results of studies published until then on the parameters of OS are not unequivocal. In published studies SOD, MDA, CAT and GPx were not studied simultaneously. We did not find any studies in which the parameters of OS we were testing would be compared depending on overweight and obesity as well as IR.

The aim of the study was to evaluate the parameters of OS in PCOS and their activity in relation to women without menstrual disorders with a normal body weight (control group). Then, MDA, SOD, CAT, and GPx were compared, differentiating between overweight and obesity, hyperandrogenemia, and IR in the PCOS group. Identification of the mechanism most involved in the protection of anti-free radicals and the sequence of involvement in the course of the development of the pathological condition were investigated.

## 2. Material and Methods

### 2.1. The Chemical and Reagents

The reagents used were from Sigma-Aldrich Poznań, Poland, and COPD Gliwice, Poland.

### 2.2. Study Group

The study included 47 women aged 18–46. Patients were hospitalized in 2017–2019 in the Department of Endocrinology, Gynecology, and Gynecological Oncology Pomeranian Medical University in Szczecin, Poland. The approval number of the study: KB-0012/87/13. Those women who were hospitalized for menstrual disorders in the form of infrequent menstruation, with PCOS diagnosed on the basis of Rotterdam Criteria [10], qualified for the study group (*n* = 26). The control group (*n* = 21) consisted of patients without menstrual disorders and without PCO in an ultrasound examination. The control group consisted of potentially healthy women recruited during screening. In all patients, ultrasound examination of the uterus and adnexa was performed (General Electric, Voluson E8 Expert, vaginal head 7.5 Mhz). Diabetes mellitus, ischemic heart disease, hypertension, patients on current and past hormonal therapy, lipid-lowering or insulin-sensitizing drugs, and antioxidant supplementation constituted the exclusion criteria in both groups (study group with PCOS and control group). Patients with PCOS were diagnosed between the 2nd and 5th day of the cycle. A history was taken in all patients and a physical examination and a hirsutism assessment were performed according to the Ferriman–Gallwey scale [26]. If the severity of symptoms was assessed at >8 points, hirsutism was diagnosed.

### 2.3. Anthropometric Measurements

In every patient, waist-hip ratio (WHR) and body mass index (BMI) were measured. The normal range of WHR is <0.8. Based on the 2016 guidelines of the American Association of Clinical Endocrinologists (AACE) and the American College of Endocrinology (ACE), BMI values in the range of 18.5–24.99 kg/m^2^ were assumed to be standard and those ≥25 kg/m^2^ were taken as indicative of excess weight and obesity [27,28,29]. Blood pressure in patients was also determined.

### 2.4. Biochemical Analyses

Fasting blood samples were collected by ulnar vein puncture. Concentration levels of FSH, LH, oestradiol, testosterone (T), androstendion (A), sex hormone binding globulin (SHBG), glucose, and insulin (fasting blood and 120 min later after an oral load of 75 g of glucose), triglycerides (TG), high-density lipoprotein (HDL), low-density lipoprotein (LDL), and total cholesterol (TChol) were established from peripheral blood serum. Two ethylenediaminetetraacetic acid (EDTA) tubes containing 7 mL of blood in total were centrifuged (1000× *g*, 4 °C, 15 min.), and plasma was separated and frozen at −80 °C.

In order to assess IR in patients, the following index was calculated: HOMA (HOMA-IR = fasting glucose concentration [mmol/L] × fasting insulin concentration [μIU/mL]/22.5; *n*: <8 [30,31,32]. IR was diagnosed on the basis of the HOMA index when its value was equal to or greater than 3.8.

The free androgen index (FAI) was calculated; T (nmol/L)/SHBG (nmol/L) × 100.

### 2.5. Analysis of Antioxidant Activity

Prior to spectrophotometric assessment of the enzymatic activity of CAT, SOD, and GPx, it was necessary to establish the contents of hemoglobin in the tested samples of hemolysates. For this purpose, the Drabkin method was applied [33].

The enzymatic activity of GPx in the erythrocyte samples was determined by the spectrophotometric method according to the Wendel method [34].

The enzymatic CAT activity in the erythrocytes was determined using a spectrophotometer according to the Aebi method [35]; SOD activity in the erythrocytes was determined using the spectrophotometric method, according to the Misra and Fridovich procedure [36].

MDA determination on a high-performance liquid chromatography (HPLC) [37] was performed using an Agilent Technologies 1200 Series using HyperSIL BDS-C18 column 3.0 × 125 mm, 3µm, and HyperSIL ODS pre-column 4 × 4 mm, 5µm.

### 2.6. Statistical Analysis

Statistical analysis was conducted using SPSS Statistics, version 13.0 (StatSoft, Cracow, Poland). Basic statistics, including mean, standard deviation, and ranges, were applied to the group characteristics. Normality of distribution was checked with the Shapiro–Wilk test. The analysis of group differences was calculated using the Mann–Whitney U test. Correlations between the analyzed quantitative variables were calculated using the rho Spearman correlation. The assumed level of significance was *p* ≤ 0.05.

## 3. Results

The mean age of the study group (*n* = 26) was 28.09 ± 6.68, and that of the control group (*n* = 21) was 32.84 ± SD 9.92. Hirsutism of >8 points was found in 7.69% (*n* = 2) and acne in 57.69% (*n* = 15) of PCOS patients. Hirsutism and acne were not found in the control group. Six patients (23.08%) from the study group were treated for infertility. The results of anthropometric tests in the group with PCOS and the control group are given in Table 1. The differences between the groups were not significant.

The values of the hormone concentration tested are shown in Table 2. A values were considerably (*p* < 0.0001) higher in patients with PCOS (3.61 ± 1.2) vs. the control group (2.3 ± 1.17). T values and FAI were also considerably (*p* < 0.002) higher in the PCOS group. SHBG values were lower in the study group, but the difference between the groups was not significant. The average Chol, LDL, HDL, and TG values in the studied groups were similar with no significant differences (Table 2).

We evaluated the OS parameters in the study and control group. Values of MDA concentration in PCOS patients were considerably higher (0.10 ± 0.02 vs. 0.08 ± 0.03). Values of CAT activity were also considerably higher in patients with PCOS (374.69 ± 72.34) vs. the control group (182.14 ± 34.85). The values of the other parameters of OS did not differ significantly between the PCOS group and the control group. The values of OS parameters are presented in Table 3.

In the group of women with PCOS, elevated androstendion values of >3.5 and/or hirsutism of >8 pts were found in 13 patients (50%); 12 patients (46.15%) were in the excessive weight and obese category. There were seven subjects (26.9%) with IR. Four patients (15.38%) were in the excessive weight and obese category with coexisting IR. There were two patients (9.5%) in the control group with hyperandrogenism. Nine patients (42.85%) were overweight or obese. Two subjects (9.5%) were diagnosed with IR. Two patients (9.5%) were overweight or obese with coexisting IR. The OS parameters were evaluated depending on IR, obesity, and hyperandrogenism (Table 4 and Table 5).

In seven PCOS patients, IR was confirmed. In these women, MDA values (mean 0.11; SD: 0.02) were significantly higher (*p*= 0.024) than in patients without IR. GPx activity showed a trend in PCOS patients (*p* = 0.067). 

In 12 obese patients with PCOS without coexisting IR, GPx activity showed a trend again (*p* = 0.075).

In four patients with PCOS and IR, coexisting obesity was found. In these women, the GPx (mean: 5.86; SD: 0.38) values were significantly higher than in patients without IR and obesity. Moreover, the trend towards SOD was noticeable.

Hyperandrogenism in the PCOS group was found in 13 women.

MDA, CAT, SOD, and GPx activity were insignificantly lower in comparison to that in the PCOS patients without hyperandrogenism (Table 4). 

Correlations between BMI values and OS parameters and between the occurrence of abdominal obesity and OS parameters in both groups of patients were examined (Table 6 and Table 7).

A significant correlation was found between hip circumference values and SOD and GPx activities in the healthy controls, respectively, R = 0.526; *p* = 0.014 and R = 0.433; *p* = 0.050 (Table 6).

Correlations between TChol, HDL, LDL, TG, and GPx activities in patients with and without PCOS were analyzed (Table 7).

In the group of patients with PCOS, there were significant reverse correlations between TChol concentrations and CAT activity (R = −0.390; *p* = 0.049), total and LDL concentrations and MDA, respectively (R = −0.437; *p* = 0.025 and R = −0.483; *p* = 0.012). In the control group, significant correlations were found between TChol LDL, and TG concentrations and GPx, respectively (R = 0.679; *p* = 0.001, R = 0.562; *p* = 0.008, and R = 0.615; *p* = 0.003). The other correlations were insignificant.

## 4. Discussion

Women with an elevated level of testosterone have a higher risk of vascular disorders [6]. In patients with PCOS, elevated OS parameters were also found to be present; hence, free oxygen radicals and OS constitute contributing factors in the development of atherosclerosis [38,39,40]. The literature assessing the presence and role of OS parameters in PCOS patients appears to be, so far, fairly inconclusive; some studies reported a reduced activity of CAT and GPx [41,42,43]; in others, elevated MDA, SOD, CAT, and GPx values were found [44,45,46,47].

MDA is one of the OS parameters and a good biomarker of OS. MDA results from lipid peroxidation of polyunsaturated fatty acids [25,48]. In our study, MDA values were significantly higher in patients with PCOS compared with healthy controls. We observed that MDA values were significantly higher in PCOS patients with IR compared with PCOS without IR, and this was independent of obesity. Some authors have suggested that IR is regarded as one of the core mechanisms by which obesity contributes to OS [11]. Others observed that the correlation of OS and IR is independent of obesity [49,50]. Kuscu et al. also demonstrated the MDA level was significantly higher in the PCOS group and it was independent of obesity, similar to our study [48]. In another study, Zhang et al. also showed that serum MDA levels in PCOS patients were significantly higher than in the control group [46]. Olusi et al. established that MDA concentration presented significantly higher in patients with BMI > 40 kg/m^2^, compared to women with standard BMI [51]. Both authors believe that in obese women, the cytoreductive activity of enzymes is reduced, and this contributes to OS. Contrary to that belief, significant changes in OS parameters in overweight and obese patients with PCOS were not observed in our study; however, there were no morbidly obese patients in our group. This may result from the fact that the main factor intensifying OS is IR, not obesity. It should be remembered that in the case of PCOS, IR is often diagnosed also in women who are not overweight or obese; therefore, it is essential that all PCOS patients, both slim and obese, be diagnosed for IR and considered for antioxidant use. IR increases the release of insulin, which in turn increases the release of ROS, which reduces the activity of antioxidants [6].

CAT, SOD, and GPx are macromolecular antioxidants (enzymes) [52]. GPx is an enzyme that protects the organism by reducing lipid hydroperoxides to their corresponding alcohols and reducing H_2_O_2_ to water. In our study, the GPx activity was similar in both PCOS and healthy controls. The GPx activity evaluation for antioxidant defense assessment in PCOS was reported in several studies. Sabuncu et al. determined oxidant and antioxidant status in women with PCOS. They demonstrated that GPx did not differ between a PCOS group and a healthy control group [44].

Baskol et al. showed similar results, demonstrating that GPx did not differ between a PCOS group and a healthy control group [53]. In contrast, Szczuko et al. observed a lower concentration of GPx in women with PCOS starting treatment, compared with the GPx activity in healthy controls [6]. We suggest that GPx is involved at every stage of the development of the pathological condition in PCOS. As a selenium-dependent enzyme, its activity may be controlled by the availability of selenium, and the supply of this component in the diet may not be sufficient [6].

In the case of patients with PCOS and IR, significant values of MDA concentrations were found compared to the PCOS group without IR. This probably suggests a different mechanism of disorders relative to OS in patients with IR and PCOS. Moreover, MDA has been associated with an increase in TChol and especially LDL; therefore, it appears that IR and an increase in LDL are the major causes of MDA mobilization.

Catalase provides protection for cells from the toxic effects of hydrogen peroxide and other derivatives. It converts H_2_O_2_ into molecular oxygen and water, without producing free radicals. In this reaction, oxygen is formed, which is used in other metabolic transformations [54]. It has been observed that in the case of diseases preceded by inflammation, the activity of CAT in the blood plasma increases. This is most likely due to its leakage from damaged cells, especially from erythrocytes [55,56]. Low activity of CAT in the blood was observed in patients with atherosclerosis and diabetes, which indicates long-lasting OS in the cells of the body of these people. H_2_O_2_ has been shown to damage pancreatic b-cells, which with CAT deficiency leads to the development of diabetes [43,57].

In our study CAT activities were significantly higher in PCOS patients, compared to the control group, and its activities were related to TChol in the PCOS group but not to a specific parameter of the lipid profile. It seems that CAT is the least sensitive parameter among the respondents in PCOS.

SOD is an enzyme and an important antioxidant defense that eliminates superoxide anions (O_2_), as a major oxygen radical. SOD catalyzes them to H_2_O_2_ and finally by GPx converted to water [58]. SOD did not differ between the studied groups, but due to the increase in IR and obesity, the trend was noticeable. We suggest that the increase in body fat leading to obesity and the increase in IR activate SOD; however, this is not an immediate reaction of the body. Murri et al. and Kuscu et al. demonstrated that SOD levels were significantly higher in a PCOS group than in the control group [8,47]. Similar results were observed by Sabuncu et al. [44]. In contrast, Seleem et al., in their study, showed that in PCOS women, the SOD level was significantly lower than in the control group [59]. These differences may be due to the treatment method and the severity of the disease.

The relationship between metabolic syndrome and cardiovascular disorders is undeniable and unquestionable. Wang et al. investigated OS parameters in patients with PCOS with and without metabolic syndrome. Metabolic syndrome, due to increased TG concentration and LDL cholesterol levels, was found to not only enhance OS but also reduce antioxidative activity [60]. In our study, we observed a similar trend in patients with PCOS; there was an inverse relationship between MDA and CAT with concentrations of TChol as well as MDA and LDL. In the control group, we observed significant correlations between TG concentration, TChol, LDL, and peroxidase activity. This confirms the thesis that PCOS with accompanying OS exponents can be an important factor predisposing patients to cardiovascular complications. Obesity is regarded as one of the risk factors for cardiovascular diseases. In our study, we found positive correlations of SOD and GPx with hip circumference only in the control group. In the PCOS group, markers of OS did not correlate with obesity-related parameters. This is supported by the fact that the major disorder in PCOS is IR, not obesity, and changes in the antioxygen system are independent of obesity. Therapeutic management strategy in patients with PCOS should, therefore, not only point towards the treatment of infertility and menstrual disorders but, through the improvement of antioxidative mechanisms, be also directed at managing metabolic disorders, including hyperinsulinemia, IR, and reduction in OS. This can be partially achieved through diet, physical exercise, and pharmacological treatment with antioxidants [8]. Divergent results of studies assessing OS parameters may indicate the existence of additional factors conditioning the antioxidant abilities of the organism. Our observations require further research because our study evaluated one of the markers of oxidative stress-MDA. The remaining parameters are an enzymatic activity of GPx, CAT, SOD.

## 5. Strengths and Limitations

A wide panel of measured parameters and clinical implementations of the study for the treatment of patients with PCOS are strong points of the research. A relatively small study sample, selected on the basis of inclusion/ exclusion criteria may be a research limitation. To confirm the validity of the findings, additional research on larger samples of subjects is thus recommended. Due to the small number of participants in the study and control groups, conclusions must be drawn with caution. Larger studies on this subject are needed to draw final conclusions.

## 6. Conclusions

It seems that an increase in hip circumference stimulates the activation of GPx and SOD and is not associated with pathological processes. The development of obesity is the beginning of a pathological process involving OS in which we observed an increase in GPx activity. If obesity is added to IR, it triggers SOD. In addition, the coexistence with IR of obesity and/or lipid profile disorders activates mechanisms associated with MDA activation and catalase. At the same time, the increase in LDL lowers MDA, and GPx is involved at every stage of the development of the pathological condition.

Therefore, IR may be the main risk factor to exposure to OS in patients with PCOS, independent of obesity. The implementation of antioxidants in PCOS therapy, in addition to drugs that improve the insulin sensitivity of tissues and physical activity, should have beneficial therapeutic effects on this group of patients.

## Figures and Tables

**Table 1:** Results of anthropometric tests in the PCOS and control group.

	Control Group *n* = 21		Study Group *n* = 26		*p*
	Mean	SD	Mean	SD	
Height (cm)	167.65	5.77	167.61	5.67	0.731
Body weight (kg)	68.95	12.92	73.67	16.19	0.314
HC (cm)	101.19	9.52	106.09	11.55	0.203
WC (cm)	82.73	12.12	85.67	16.7	0.855
WHR	0.815	0.063	0.801	0.098	0.356
BMI (kg/m^2)^	24.53	4.37	26.57	6.68	0.362

HC—Hip circumference; WC—Waist circumference; WHR—waist-hip ratio; BMI—body mass index; *p—p* value.

**Table 2:** Concentration values of tested hormones and FAI in the PCOS and control groups.

Parameter	Control Group *n* = 21		Study Group *n* = 26		*p*
	Mean	SD	Mean	SD	
**T [ng/mL]**	0.318	0.169	0.50	0.24	**0.002**
SHBG [nmol/L]	82.16	74.02	49.64	22.7	0.113
A [ng/mL]	2.3	1.17	3.61	1.20	**0.0001**
FAI	0.61	0.45	1.15	0.63	**0.002**
LDL [mg/dL]	108.09	35.27	112.41	39.9	0.814
HDL [mg/dL]	65.66	15.54	58.14	16.4	0.118
TG [mg/dL]	98.46	75.38	107.36	96.91	0.846
TChol [mg/dL]	180.79	39.3	170.43	42.03	0.313

T—testosterone; SHBG—sex hormone binding globulin; A—androstendion; FAI—free androgen index; LDL—low-density lipoprotein; HDL—high-density lipoprotein; TG—triglycerides; TChol—total cholesterol; *p—p* value. bold—significant differences.

**Table 3:** The values of oxidative stress parameters.

Parameter	Control Group (*n* = 21)		Study Group (*n* = 26)		
	Mean	SD	Mean	SD	*p*
MDA [uM]	0.08	0.03	0.10	0.02	**0.003**
CAT [k/gHb]	182.1	34.85	374.7	72.34	**2 × 10^−6^**
SOD [A/gHb]	1695.9	125.2	1701.1	270.5	0.500
GPx [U/gHb]	5.06	0.75	4.95	0.71	0.612

MDA—malonylodialdehyde; CAT—catalase; SOD—superoxide dismutase; GPx—glutathione peroxidase; *p—p* value. bold—significant differences.

**Table 4:** Comparison of MDA, SOD, CAT catalase, and GPx glutathione peroxidase among women with PCOS (SG) depending on insulin resistance (A), obesity (B), excess weight and obese with insulin resistance (C), and hyperandrogenism (D).

A.	Without Insulin Resistance (*n* = 19)		Insulin Resistance (*n* = 7)		
Parameter	Mean	SD	Mean	SD	*p*
MDA [uM]	0.09	0.01	0.11	0.02	**0.024**
CAT [k/gHb]	365.26	70.46	400.29	76.56	0.355
SOD [A/gHb]	1694.33	271.76	1719.67	287.76	0.773
GPx [U/gHb]	4.79	0.66	5.36	0.72	0.067
**B.**	**Without Obesity (*n* = 14)**		**Obese (*n* = 12)**		
**Parameter**	**Mean**	**SD**	**Mean**	**SD**	***p***
MDA [uM]	0.10	0.02	0.10	0.02	0.738
CAT [k/gHb]	386.14	57.61	361.33	87.23	0.396
SOD [A/gHb]	1641.34	256.84	1770.93	280.17	0.123
GPx [U/gHb]	4.72	0.68	5.21	0.67	0.075
**C.**	**Without Obesity and Insulin Resistance (*n* = 22)**		**Obese with Insulin Resistance (*n* = 4)**		
**Parameter**	**Mean**	**SD**	**Mean**	**SD**	***p***
MDA [uM]	0.10	0.01	0.12	0.02	0.127
CAT [k/gHb]	368.00	66.99	411.50	100.27	0.303
SOD [A/gHb]	1669.93	272.65	1872.84	207.47	0.082
GPx [U/gHb]	4.78	0.62	5.86	0.38	**0.003**
**D.**	**Without Hyperandrogenism (*n* = 13)**		**With Hyperandrogenism (*n* = 13)**		
**Parameter**	**Mean**	**SD**	**Mean**	**SD**	***p***
MDA [uM]	0.10	0.02	0.10	0.02	0.442
CAT [k/gHb]	388.77	70.93	360.62	73.76	0.412
SOD [A/gHb]	1717.68	260.47	1684.62	289.87	0.837
GPx [U/gHb]	4.98	0.76	4.91	0.68	0.795

MDA—malonylodialdehyde; CAT—catalase; SOD—superoxide dismutase; GPx—glutathione peroxidase; *p*—*p* value; bold—significant differences.

**Table 5:** Comparison of MDA, SOD, CAT catalase, and GPx glutathione peroxidase among women without PCOS depending on obesity, insulin resistance, and hyperandrogenism.

A	Without Insulin Resistance (*n* = 19)		Insulin Resistance (*n* = 2)		
Parameter	Mean	SD	Mean	SD	*p*
MDA [uM]	0.08	0.03	0.08	0.00	0.952
CAT [k/gHb]	182.32	35.39	180.50	41.72	0.857
SOD [A/gHb]	1698.32	128.16	1673.00	130.11	0.905
GPx [U/gHb]	4.94	0.70	6.13	0.16	**0.031**
**B**	**Without Obesity(*n* = 12)**		**Obese (*n* = 9)**		
**Parameter**	**Mean**	**SD**	**Mean**	**SD**	***p***
MDA [uM]	0.09	0.04	0.08	0.02	0.972
CAT [k/gHb]	180.75	41.07	184.00	26.66	0.696
SOD [A/gHb]	1688.92	138.00	1705.22	113.40	0.546
GPx [U/gHb]	4.91	0.61	5.25	0.92	0.330
**C**	**Without Insulin Resistance and Obesity (*n* = 19)**		**Insulin Resistance and Obesity (*n* = 2)**		
**Parameter**	**Mean**	**SD**	**Mean**	**SD**	***p***
MDA [uM]	0.08	0.03	0.08	0.00	0.952
CAT [k/gHb]	182.32	35.39	180.50	41.72	0.857
SOD [A/gHb]	1698.32	128.16	1673.00	130.11	0.905
GPx [U/gHb]	4.94	0.70	6.13	0.16	**0.031**
**D**	**Without Hyperandrogenism (*n* = 19)**		**Hyperandrogenism (*n* = 2)**		
**Parameter**	**Mean**	**SD**	**Mean**	**SD**	***p***
MDA [uM]	0.09	0.03	0.05	0.01	**0.036**
CAT [k/gHb]	182.42	35.73	179.50	36.06	0.952
SOD [A/gHb]	1689.74	127.56	1754.50	115.26	0.549
GPx [U/gHb]	5.00	0.78	5.56	0.05	0.338

MDA—malonylodialdehyde; CAT—catalase; SOD—superoxide dismutase; GPX—glutathione peroxidase; *p*—*p* value; bold—significant differences.

**Table 6:** Correlations between BMI, WHR values, and OS parameters and between the occurrence of abdominal obesity and OS parameters in the PCOS and control groups.

Variable Pair	PCOS *n* = 26		Control *n* = 21	
	R Spearman	*p*-Value	R Spearman	*p*-Value
WHR & MDA	0.221652	0.276	0.096135	0.678
WHR & CAT	−0.107748	0.600	−0.251380	0.271
WHR & SOD	0.139754	0.495	−0.144899	0.530
WHR & GPx	−0.015906	0.938	0.321962	0.154
HIP & MDA	−0.297272	0.140	0.366060	0.102
HIP & CAT	0.096686	0.638	−0.020156	0.930
HIP & SOD	0.343699	0.085	**0.526504**	**0.014**
HIP & GPx	−0.006526	0.974	**0.433171**	**0.049**
BMI & MDA	−0.144323	0.481	0.359319	0.143
BMI & CAT	0.090629	0.659	−0.170367	0.499
BMI & SOD	0.371473	0.061	0.283058	0.255
BMI & GPx	−0.022914	0.911	0.340909	0.166

Aggregated results. R Spearman—Spearman rank order correlation; BMI—body mass index; WHR waist–hip ratio; MDA—malonylodialdehyde; CAT—catalase; SOD—superoxide dismutase; GPX3—glutathione peroxidase; HIP—hip circumference *p*—*p* value; bold—significant differences.

**Table 7:** Correlations between HDL, LDL, TG, TChol values, and oxidative stress parameters and between the occurrence of abdominal obesity and oxidative stress parameters in the PCOS group (SG) and control groups (CG).

Variable Pair	PCOS *n* = 26		Control *n* = 21	
	R Spearman	*p*-Value	R Spearman	*p*-Value
TChol & MDA	**−0.437415**	**0.025**	0.149351	0.518
TChol & CAT	**−0.390561**	**0.049**	−0.010390	0.964
TChol & SOD	−0.266461	0.188	0.102631	0.658
TChol & GPx	0.049932	0.809	**0.679441**	**0.001**
HDL & MDA	−0.053010	0.797	0.032468	0.889
HDL & CAT	−0.299248	0.138	−0.129870	0.575
HDL & SOD	−0.386181	0.051	0.215005	0.349
HDL & GPx	−0.102941	0.617	−0.363754	0.105
LDL & MDA	**−0.483926**	**0.012**	0.028571	0.902
LDL & CAT	−0.167579	0.413	0.122078	0.598
LDL & SOD	−0.123140	0.549	−0.186424	0.418
LDL & GPx	0.127565	0.536	**0.562520**	**0.008**
TG & MDA	−0.114931	0.576	0.284416	0.211
TG & CAT	0.058834	0.775	0.393506	0.078
TG & SOD	0.127609	0.534	−0.399480	0.073
TG & GPx	0.053703	0.794	**0.615135**	**0.003**

Aggregated results. Spearman rank order correlation; MDA—malonylodialdehyde; CAT—catalase; SOD—superoxide dismutase; GPx—glutathione peroxidase; LDL—low-density lipoprotein; HDL—high-density lipoprotein; TG—tryglycerides; TChol—total cholesterol; *p*—*p* value; bold—significant differences.

## Data Availability

The data are available on request.

## References

[1] Diamanti-Kandarakis E., Kouli C.R., Bergiele A.T., Fulandra F.A., Tsianatelli T.C., Spina G.G., Zapandi E.D., Bartzis M.I. (1999). A survey of the polycystic ovary syndrome in the Greek island of Lesbos: Hormonal and metabolic profile. J. Clin. Endocrinol. Metab..

[2] Sanchon R., Gambineri A., Alpanes M., Martinez-Garcia M.A., Pasquali R., Escobar-Morreale H.F. (2012). Prevelence of functional disorders of androgenn excess in unselected premenopausal women: A study in blood donors. Hum. Reprod..

[3] McCartney C.R., Marshall J.C. (2016). Clinical practice. Polycystic ovary syndrome. N. Engl. J. Med..

[4] Acmaz G., Cınar L., Acmaz B., Aksoy H., Kafadar Y.T., Madendag Y., Ozdemir F., Sahin E., Muderris I. (2019). The Effects of Oral Isotretinoin in Women with Acne and Polycystic Ovary Syndrome. Biomed Res. Int..

[5] Escobar-Morreale H.F., Villuendas G., Botella-Carretero J., Alvarez-Blasco F., Sancon R., Luque-Ramirez M., San Millan J.L. (2006). Adiponectin and resistin in PCOS: A clinical, biochemical and molecular genetic study. Hum. Reprod..

[6] Szczuko M., Zapałowska-Chwyć M., Drozd R. (2019). A Low glycemic Index Decreases Inflammation by Increasing the concentration of uric acid and the activity of glutathione peroxidase (GPx3) in Patients with Polycystic Ovary Syndrome (PCOS). Molecules.

[7] Dumesic D.A., Lobo R.A. (2013). Cancer risk and PCOS. Steroids.

[8] Murri M., Luque-Ramirez M., Insenser M., Ojeda-Ojeda M., Escobar-Morreale H.F. (2013). Circulating markers of oxidative stress and polycystic ovary syndrome (PCOS): A systematic review and meta-analysis. Hum. Reprod. Update.

[9] Bentley-Lewis R., Seely E., Dunaif A. (2011). Ovarian Hypertension: Polycystic Ovary Syndrome. Endocrinol. Metab. Clin. N. Am..

[10] The Rotterdam ESHRE/ASRAM-sponsored PCOS Consensus Workshop Group (2004). Revised 2003 consensus on diagnostic criteria and long-term health risks related to polycystic ovary syndrome. Hum. Reprod..

[11] Zuo T., Zhu M., Xu W. (2016). Roles of Oxidative Stress in Polycystic Ovary Syndrome and Cancers. Oxidative Med. Cell. Longev..

[12] Facchini F.S., Hua N.W., Raeven G.M., Stoohs R.A. (2000). Hiperinsulinemia: The missing link among oxidative stress and age-related diseases?. Free Radic. Biol. Med..

[13] Duica F., Danila C.A., Boboc A.E., Antoniadis P., Condrat C.E., Onciul S., Suciu N., Creţoiu S.M., Varlas V.N., Creţoiu D. (2021). Impact of increased Oxidative Stress on cardiovascular diseases in women with polycystic ovary syndrome. Front. Endocrinol..

[14] Dumesic D.A., Richards J.S. (2013). Ontogeny of the ovary in polycystic ovary syndrome. Fertil. Steril..

[15] Webber L.J., Stubbs S., Stark J., Trew G.H., Margara R., Hardy K., Franks S. (2003). Formation and early development of follicles in the polycystic ovary. Lancet.

[16] Spritzer P.M., Lecke S.B., Satler F., Morsch D.M. (2015). Adipose tissue dysfunction, adipokines, andlow- grade chronic inflammation in polycystic ovary syndrome. Reproduction.

[17] Szczuko M., Zapałowska-Chwyć M., Maciejewska D., Drozd A., Starczewski A., Stachowska E. (2016). High glycemic index diet in PCOS patients, The analysis of IGF I and TNF-a pathways in metabolic disorders. Med. Hypotheses.

[18] Evans J.L., Goldfine I.D., Maddux B.A., Grodsky G.M. (2003). Are oxidative stress-activated signaling pathways mediators of insulin resistance and beta-cell dysfunction?. Diabetes.

[19] Turrens J.F. (2003). Mitochondrial formation of reactive oxygen species. J. Physiol..

[20] Valko M., Leibfritz D., Moncol J., Cronin M.T., Mazur M. (2006). Free radicals, metals and antioxidants in oxidative stress-induced cancer. Chem. Biol. Interact..

[21] Pannala A.S., Rice-Evans C., Sampson J., Singh S. (1998). Interaction of peroxynitrite with carotenoids and tocopherols within low density lipoprotein. FEBS Lett..

[22] Heales S.J.R. (2001). Catalase deficiency, diabetes and mitochondrial function. Lancet.

[23] Goth L., Rass P., Pay A. (2004). Catalase enzyme mutations and their association with diseases. Mol. Diagn..

[24] Dubey P., Reddy S., Boyd S., Bracamontes C., Sanchez S., Chattopadhyay M., Dwivedi A. (2021). Effect of Nutritional Supplementation on Oxidative Stress and Hormonal and Lipid Profiles in PCOS-Affected Females. Nutrients.

[25] Vincent H.K., Taylor A.G. (2005). Biomarkers and potential mechanism of obesity-induced oxidant stress in humans. Int. J. Obes..

[26] Ferriman D., Gallwey J.D. (1961). Clinical assessment of body hair growth in women. J. Clin. Endocrinol. Metab..

[27] Garvey W.T., Mechanick J.I., Brett E.M., Garber A.J., Hurley D.L., Jastreboff A.M., Nadolsky K., Pessah-Pollack R., Plodkowski R. (2016). American Association of Clinical Endocrinologists and American College of Endocrinology Comprehensive Clinical Practice Guidelines for Medical Care of Patients with Obesity. Endocr. Pract..

[28] Olszanecka-Glinianowicz M. (2017). Leczenie farmakologiczne otyłości w świetle aktualnych wytycznych American Association of Clinical Endocrinologists and American College of Endocrinology 2016. Med. Prakt..

[29] Nuttall F.Q. (2015). Body Mass Index–Obesity, BMI, and Health: A Critical Review. Nutr. Res..

[30] Marques-Vidal P., Mazoyer E., Bongard V., Gourdy P., Riudavets J.B., Drouet L., Ferrieres J. (2002). Prevalence of insulin resistance syndrome in Southwestern France and its relationship with inflammatory and haemostatic markers. Diabetes Care.

[31] Esteghamati A., Ashraf H., Khalilzadeh O., Zandieh A., Nakhjavani M., Rashidi A., Haghaali M., Asgari F. (2010). Optimal cut-off of homeostasis model assessment of insulin resistance (HOMA-IR) for the diagnosis of metabolic syndrome: Third national surveillance of risk factors of non-communicable diseases in Iran (SuRFNCD-2007). Nutr. Metab..

[32] Tang Q., Li X., Song P., Xu L. (2015). Optimal cut-off values for the homeostasis model assessment of insulin resistance (HOMA-IR) and pre-diabetes screening: Developments inresearch and prospects for the future. Drug Discov. Ther..

[33] Drabkin D.L. (1949). The standardization of hemoglobin measurement. Am. J. Med. Sci..

[34] Wendel A. (1981). Glutathione peroxidase. Methods Enzym..

[35] Aebi H. (1984). Catalase in vitro. Met. Enzymol..

[36] Misra H.P., Fridovich I. (1972). The role of superoxide anion in the autoxidation of epinephrine and a simple assay for superoxide dismutase. J. Biol. Chem..

[37] Templari J., Kon S.P., Milligan T.P., Newman D.J., Raftery M.J. (1999). Increased plasma malondialdehyde levels in glomerular disease as determined by a fully validated HPLC method. Nephrol. Transpl..

[38] Davey M.W., Stals E., Panis B., Keulemans J., Swennen R.L. (2005). High-throughput determination of malondialdehyde in plant tissues. Anal. Biochem..

[39] Dokras A., Jagasia D., Maifeld M., Sinkey C.A., VanVoorhis B.J., Haynes W.G. (2006). Obesity and insulin resistance but not hyperandrogenism mediates vascular dysfunction in women with policytsic ovary syndrome. Fertil. Steril..

[40] Gonzales F., Rote N., Minum J., Kirwan J.P. (2006). Reactive androgen species induced oxidative stress in the developemnet of insulin resistance and hyperandrogenism in polycystic ovary syndrome. J. Clin. Endocrinol. Metab..

[41] Radomski D., Orzechowska A., Barcz E. (2007). Presents conceptions of ethiopatogenesis of policystic ovary syndrome. Gynecol. Pol..

[42] Enechukwu C.I., Onuegbu A.J., Olisekodiaka M.J., Eleje J.U., Ikechebelu J.I., Ugboaja J.O., Amah U.K., Okwara J.E., Igwegbe A.O. (2019). Oxidative stress markers and lipid profiles of patients with polycystic ovary syndreme in Nigerian teritiary hospital. Obstet. Gynecol. Sci..

[43] Dos Santos A.C.S., Azevedo G.D., Lemos T. (2016). The influence of oxidative stress in infalmamatory process and insulin resistance in obese women with polycystic ovary syndrome. Transi Biomed.

[44] Jeedlani H., Ganie M.A., Masood A., Amin S., Kawa I.A., Fatima Q., Manzoor S., Parvez T., Naikoo N.A., Rashid F. (2009). Assessment of PON1 activity and circulating TF levels in relation to BMI, testosterone, HOMA-IR, HDL-C, LDL-C, CHO, SOD activity and TAC in women with PCOS: An observational study. Diabetes Metab. Syndr..

[45] Sabuncu T., Vural H., Harma M. (2001). Oxydative stress in polycystic ovary syndrome and its contribution to the risk of cardiovascular disease. Clin. Biochem..

[46] Zhang D., Luo W.Y., Liao H., Wang C.F., Sun Y. (2008). The effects of oxidative stress to PCOS. Sichuan Da Xue Xue Bao Yi Xue Ban.

[47] Desai V., Prasad N.R., Manohar S.M., Sachan A., Narasimmha S.R., Bitla A.R. (2014). Oxydative stress in non-obese women with polycystic ovary syndrome. J. Clin. Diagn. Res..

[48] Kuscu N.K., Var A. (2009). Oxidative stress but not endothelial dysfunction exist in non-obese young group of patients with polycystic ovary syndrome. Acta Obstet. Et Gynecol. Scand..

[49] Mohammadi M. (2019). Oxidative stress and polycystic ovary syndrome: A Brief Review. Int. J. Prev. Med..

[50] Savic-Radojevic A., Antic I.B., Coric V., Bjekic-Macut J., Radic T., Zarkovic M., Djukic T., Pljesa-Ercegovac M., Panidis D., Katsikis I. (2015). Effect of hyperglycemia and hyperinsulinemia on glutathione peroxidase activity in non-obese women with polycystic ovary syndrome. Hormone.

[51] Olusi S. (2002). Obesity is an independent risk factor for plasma lipid peroxidation and depletion of erythrocyte cytoprotectic enzymes in humans. Int. J. Obes. Relat. Metab. Disord..

[52] Karpińska A., Gromadzka G. (2013). Oxidative stress and natural antioxidant mechanism: The role in neuroregeneration. From molecular mechanism to therapeutic strategies. Postepy Hig. Med. Dosw..

[53] Baskol G., Aygen E., Erdem F., Caniklioglu A., Narin F., Sahin Y., Kaya T. (2012). Assessment of paraoxonase 1, xanthine oxidase and glutathione peroxidase activities, nitric oxide and thiol levels in women with polycystic ovary syndrome. Acta Obstet. Et Gynecol. Scand..

[54] Chelikani P., Fita I., Loewen P.C. (2004). Diversity of structures and properties among catalases. Cell. Mol. Life Sci..

[55] Scibior D., Czeczot H. (2006). Catalase: Structure, properties, functions. Post Hig. Med. Dosw..

[56] Goth L., Lenkey A., Bigler W.N. (2001). Blood catalase defi ciency and diabetes in Hungary. Diabetes Care.

[57] Goth L., Eaton J.W. (2000). Hereditary catalase defi ciencies and increased risk of diabetes. Lancet.

[58] Droge W. (2002). Free radicals in the physiological control of cell function. Physiol. Rev..

[59] Seleem A.K., El Refaeey A.A., Shaalan D., Sherbiny Y., Badaway A. (2014). Speroxide dismutase in polycystic ovary syndrome patients undergoing intracytoplasmic sperm injection. J. Assist. Eprod. Genet..

[60] Wang H., Ruan X., Li Y., Cheng J., Mueck A.O. (2019). Oxidative stress indicators in Chinese women with PCOS and correlation with features of metabolic syndrome and dependency on lipid patterns. Arch. Gynecol. Obstet..

